# Adaptation and Convergent Evolution within the *Jamesonia*-*Eriosorus* Complex in High-Elevation Biodiverse Andean Hotspots

**DOI:** 10.1371/journal.pone.0110618

**Published:** 2014-10-23

**Authors:** Patricia Sánchez-Baracaldo, Gavin H. Thomas

**Affiliations:** 1 School of Geographical Sciences, University of Bristol, Bristol, United Kingdom; 2 Department of Animal and Plant Sciences, University of Sheffield, Sheffield, South Yorkshire, United Kingdom; Montreal Botanical Garden, Canada

## Abstract

The recent uplift of the tropical Andes (since the late Pliocene or early Pleistocene) provided extensive ecological opportunity for evolutionary radiations. We test for phylogenetic and morphological evidence of adaptive radiation and convergent evolution to novel habitats (exposed, high-altitude páramo habitats) in the Andean fern genera *Jamesonia* and *Eriosorus.* We construct time-calibrated phylogenies for the *Jamesonia-Eriosorus* clade. We then use recent phylogenetic comparative methods to test for evolutionary transitions among habitats, associations between habitat and leaf morphology, and ecologically driven variation in the rate of morphological evolution. Páramo species (*Jamesonia*) display morphological adaptations consistent with convergent evolution in response to the demands of a highly exposed environment but these adaptations are associated with microhabitat use rather than the páramo *per se*. Species that are associated with exposed microhabitats (including *Jamesonia* and *Eriorsorus*) are characterized by many but short pinnae per frond whereas species occupying sheltered microhabitats (primarily *Eriosorus*) have few but long pinnae per frond. Pinnae length declines more rapidly with altitude in sheltered species. Rates of speciation are significantly higher among páramo than non-páramo lineages supporting the hypothesis of adaptation and divergence in the unique Páramo biodiversity hotspot.

## Introduction

The Andes are one of the most species-rich biodiversity hotspots in the world [1] with an estimated 45,000 plant species, 44% of which are endemic [2]. As high elevation ecosystems known as páramos emerged during the last major uplifting of the Northern Andes [3]–[5], during the Pliocene and/or Pleistocene (2–4 Ma) new ecological opportunities became available for species radiations to occur [6]–[8]. Recent studies suggest that average speciation rates in páramos exceed those of other major plant diversity hotspots [9]. Notable examples of recent Andean radiating lineages include the extremely rapid speciation of the *Lupinus* clade [10], and adaptive radiations of *Valeriana* and *Gentianella* [10]–[12]. These radiations contribute to the high levels of endemism in the Northern Andean páramo ecosystems, which contribute>60% of the total plant species richness [7], [10], [13]–[15]. High levels of endemism are likely associated with glacial cycles during the Pleistocene [3]–[5]. During this period, the reduction and fragmentation of the páramo into smaller habitat islands during interglacial periods [16] may have contributed to periods of isolation. The fragmented and isolated distribution of páramo habitats is considered akin to island systems and may have enhanced the potential for species diversification [10].

Plant radiations in páramos have several possible biogeographic origins that can broadly be categorised as temperate (both Northern and Southern hemisphere) or tropical montane forest. Northern hemisphere temperate origins are perhaps the dominant source for the rapid origination and recent diversification of numerous endemic plant groups (e.g. *Draba, Gentianella*, *Lupinus, Valeriana*, and *Viburnum*; [16]). While herbaceous elements mainly diversify in páramo, montane forest tree lineages generally failed to diversify [10], [17]. Colonisation from Southern hemisphere temperate regions include *Azorella, Ourisia*, *Calceolaria* and *Puya* but overall clades with southern origins seem to have contributed less to floral diversity in the páramo than clades from the Northern hemisphere [15], [18], [19].

Because páramos are characterised by extreme environmental conditions such as strong winds, high levels of insolation, and cool temperatures ranging daily from −2°C to 12°C [7], [20], [21], the success of colonising floras from temperate regions has been attributed to potential exploitation of island-like ecological opportunities by lineages already well adapted to the cooler, more exposed habitats encountered in the high altitude páramo [10]. Examples of morphological adaptations to high altitude include microphyllous, pubescent and sclerophyllous leaves as well as growth-forms such as rosette plants, cushion plants, dwarf shrubs and geophytes [7]. In contrast, lineages with a tropical montane origin are unlikely to be pre-adapted to novel high-altitude habitats. Indeed, few studies have shown plant groups evolving traits *in situ* at high elevations [18]. Although some páramo radiations, such as the species rich genus *Espeletia* in the Asteraceae [6], [22] and *Huperzia* [23] likely had origins in the surrounding montane forest [6], [23], little is known about patterns of morphological evolution or diversification associated with transitions to high altitude. Here we test for morphological adaptation to, and divergence at, high altitudes in the *Jamesonia-Eriosorus* complex of Andean ferns.

**Figure 1:**
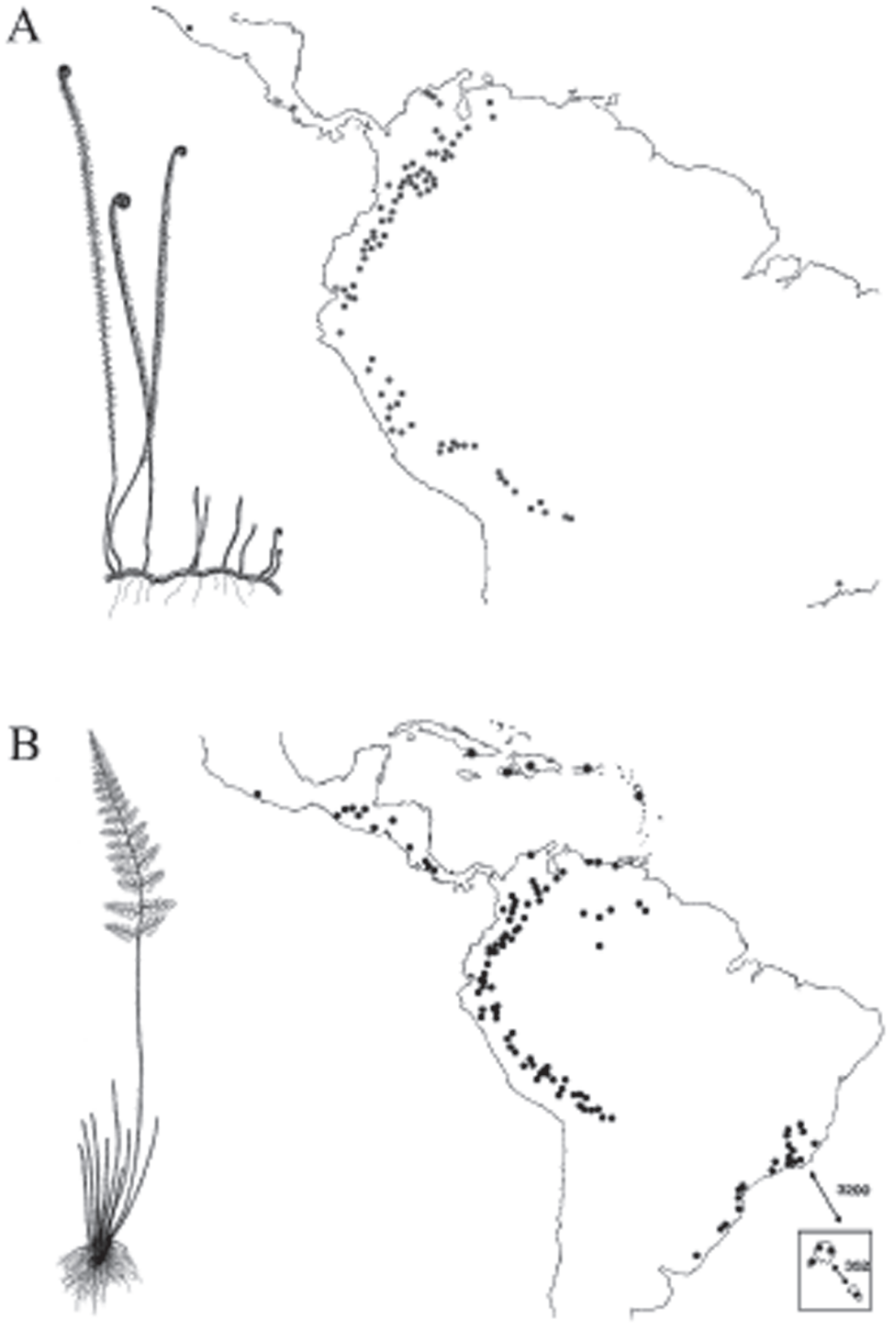
Distribution of known locations of *Jamesonia* (A) and *Eriosorus* (B) [26], [27]. The fern diagrams represent the generalized frond architecture of *Jamesonia* and *Eriosorus*. *Jamesonia* exhibit indeterminate growth (retention of fiddle head) with a high number of pinnae per frond; in contrast, the *Eriosorus* have fully developed with approximately 13 to 14 pinnae per frond with the exception of *Eriosorus flexuosus*.

The *Jamesonia-Eriosorus* complex (Fig. 1) has a probable Southern hemisphere Brazilian origin [24], [25]. The clade consists of two genera: the paraphyletic *Eriosorus* and polyphyletic *Jamesonia* that have expanded into cloud forest and páramo ecotones throughout the Northern and Central Andes [24], [25]. *Jamesonia* are typically found in páramo or exposed parts of the sub-páramo at altitudes ranging from ca. 1500 to 5000 metres. In contrast, *Eriosorus* [26] species are mainly found in cool and moist highlands such as cloud forest and in sheltered and shady microhabitats within the sub-páramo and páramo at altitudes ranging from ca. 600 to 4100 m with most species occurring above 2200 m. Previous studies of the *Jamesonia-Eriosorus* complex focused on phylogeny, specifically, resolving their phylogenetic affinities within the subfamily Taenitidoideae [25], and on the relationship between biogeography and phylogeny of the complex itself [24]. Furthermore, Sánchez-Baracaldo [24] hypothesized that *Jamesonia* species have a distinctive morphology characterized by indeterminate growth (not fully developed leaves) and an increased number of pinnae per frond that was likely favoured in the extreme environmental conditions prevailing in páramo ecosystems. Other potential adaptations include coriaceous pinnae, xeromorphic leaves, and pubescence [27]. However, whether these adaptations are associated with páramo specifically, with exposed rather than sheltered microhabitats, or more generally to upward shifts along an altitudinal gradient is untested.

In this study phylogenetic analyses confirm three Andean clades with high levels of convergent evolution in which *Jamesonia* species evolved independently likely adapting to the extreme environmental characteristics of páramo habitats [24]. Bayesian molecular clock analyses point to a Pleistocene origin for the three main lineages of *Jamesonia-Eriosorus* into both montane and páramo habitats with notably higher speciation rates in the páramo. We test whether putative morphological adaptations to exposed environments represent responses to altitudinal gradients, microhabitat (exposed vs. sheltered) or responses to the unique challenges of páramo ecosystems.

## Materials and Methods

### Ethics statement

None of the *Jamesonia* and *Eriosorus* species are or were endangered or protected species. All samples were collected during 1998–99. All specimens studied were imported into the United States under a CITES permit (the Convention on International Trade in Endangered Species of Wild Fauna and Flora) granted to the University Herbaria, at UC Berkeley (Director Prof Brent Mishler). Plant specimens were measured before they were mounted in to herbarium specimens. The main collection is housed in the University Herbaria at UC Berkeley.

Venezuela: Plant collections were made in collaboration with Enrique La Marca from Universidad de Los Andes in Merida. The Ministerio de Ambiente granted the permit in Venezuela.

Colombia: All samples were collected in collaboration with the Instituto de Biodiversidad Alexander von Humboldt (Direction at the time Dr Cristián Samper). The Ministerio de Ambiente, Bogota, granted the permit in Colombia.

Ecuador: All collections were made in collaboration with Dr Hugo Navarrete from the Herbario de la Universidad Catolica, Ecuador. The Ministerio de Ambiente, Quito, granted the permit in Ecuador.

Peru: All collections were made in collaboration Asuncion Cano from El Museo de Historia Natural, Lima. The Ministerio de Ambiente, Lima, granted the permit in Peru.

Additional samples from Bolivia were sent by Jasivia Gonzales who at the time was working at Herbario Nacional de Bolivia and samples from Brazil were sent by Jefferson Prado and working at the Instituto Botanico, Sao Paolo, Brazil.

### Ecological and morphological data

Current taxonomic treatments [28] list 59 species of *Jamesonia* and *Eriosorus*. However, recent phylogenetic studies [29] show that three species of *Eriosorus* (*E. myriophylla*, *E. schwackeana*, and *E. areniticola*) are more closely to other taenitidoid genera and propose a new genus name, *Tryonia*, for these species along with a fourth species, *E. sellowianus*. *Eriosorus sellowianus* has not yet been phylogenetically sampled but has previously been treated as a subspecies of *E. schwackeana*. We follow the phylogenetic revision resulting in a total of 55 species. For the majority of our analyses we use a subset of species but defining the total richness of the clade is important for our analyses of state dependent diversification (see the section *BiSSE: state dependent diversification* below for details).

We categorised species as present (0) or absent (1) from páramo. We scored species as present in the páramo if they occur in any of three broad páramo habitat zones based on overall altitude, vegetation structure and geographic location: 1) super-páramo (4000–5000 m) is the ecotone between permanent snow and grass páramo below and consists of coarse vegetation growing on rocky scree; 2) grass páramo (3500–4100 m) has continuous vegetation, mainly tussock or bunch grasslands; and 3) sub-páramo (2800/3000–3500 m) is the ecotone between grass páramo and montane forest and is the most floristically diverse zone [21]. Using the above description a summary of *Jamesonia* and *Eriosorus* habitats are found in Table S1. We based categorisation on field observations and monographs for the genera [26], [27].

For 25 species we also categorise species according microhabitat based on field observations by PSB from 68 localities. We classified microhabitat as sheltered (e.g., wet or moist shaded forest borders, shaded places at edge of boulders or in caves) or exposed (e.g., among rocks and/or bare soil, open fields among grasses, on very exposed slopes and cliffs); see Table S1 for a more detailed list of microhabitats. Each locality was classified independently. The localities span Costa Rica, Venezuela, Colombia, Ecuador, Peru, Bolivia and Brazil (Fig. 1).

We obtained morphological and altitudinal data for specimens from 55 out of 68 localities, sampling three to four plant specimens per locality. We measured two characters: 1) the number of pinnae along fronds in sexually mature plants (defined as those bearing sporangia), and 2) pinna length (millimetres) of the middle frond. We have chosen these morphological traits since they are variable and likely ecologically significant and we hypothesised that their variation evolved in response to exposed environmental conditions typically found in páramo.

### Sequence data and phylogenetic analyses

We re-analysed previously published [24] nucleotide data from samples taken from each of the 68 localities from the nuclear external transcribed spacer (ETS) within the 18S–26S nuclear ribosomal DNA (1152 base pairs), and the plastid *rps4* (576 bp) and intergenic spacer *rps4-trnS* (415 bp). These are rapidly evolving markers that have previously been used to investigate taxonomic relationships at low taxonomic levels [30], [31]. The same data set was previously analysed using parsimony [24]; in our reanalysis we use Bayesian relaxed clock methods to simultaneously estimate tree topology and divergence times. The data were aligned by eye with Se-Al v 2.0a11 [32]. We determined the most appropriate partitioning scheme and substitution models among loci using PartitionFinder [33]. We allowed PartitionFinder to search all possible combinations form a total of five possible partitions (ETS, *rps4* divided into codon positions, and *rps4-trnS*). The selected scheme was a TrN+Γ model for the intergenic spacer *rps4-trnS*, a K80+I model for the coding plastid *rps4* region (the best scheme combined the three codon positions), and a TVM+Γ model for ETS.

We used BEAST v. 1.8.0 [34] to reconstruct and date phylogenetic trees using a Bayesian uncorrelated relaxed molecular clock [35]. We calibrated the tree simultaneously with tree estimation using a log-normal uncorrelated relaxed clock [35], a Yule calibration prior [36], and a single fossil constraint. While there are reports for both *Jamesonia* and *Eriosorus* spores in the geological record, there are no morphological characters that can readily tell them apart and so assigning specific spores to specific nodes in the tree for calibration is not possible. We therefore used the first appearance of a spore considered to be *Eriosorus* to calibrate the root of the tree. The spore is dated at 3.5 Ma with an origin from lower elevations [3], [37]. We treated this as a minimum age constraint and applied an exponential prior (offset = 3.5, mean = 0.5006). This sets 95% of the prior distribution between 3.5 and 5 Ma, consistent with an origin coincident with the emergence of the páramo. Setting prior distributions on node ages is challenging and due to the lack of robust data with which to estimate a soft maximum constraint, we are cautious in interpretation of the exact timings for major divergences in the *Jamesonia-Eriosorus* clade. We ran four independent analyses, each with 50,000,000 generations. We used Tracer v1.5.0 [38] to assess convergence and mixing of parameters. For all subsequent analyses we use a sample of 1000 trees from the posterior distribution to account for phylogenetic uncertainty.

### Predictors of morphological evolution

To visually assess whether *Jamesonia* and *Eriosorus* morphology clusters phylogenetically or by habitat we plotted the number of leaves per frond against pinnae length using the phylomorphospace method [39] implemented in the R package phytools [40]. We then tested the relationship between morphology and habitat and altitude using phylogenetic generalized least squares (pgls) in the R package caper [41]. We fitted the following four sets of explanatory variables to each of pinnae length and leaves per frond: (1) altitude; (2) habitat; (3) altitude + habitat; (4) altitude x habitat. All pgls models were conducted while estimating the strength of phylogenetic signal in the model residuals using Pagel's λ [42], [43] and were conducted on full trees. Each analysis was applied to a posterior sample of 1000 trees. We compared the fit of the models using the small-sample Akaike Information Criterion (AICc) and Akaike weights for each tree sample and then took the average AICc and Akaike weights across all 1000 trees to infer the overall best model. We averaged the parameter estimates for the best model across all 1000 trees.

### BiSSE: state dependent diversification

We used the Binary State Speciation and Extinction model (BiSSE; [44], [45] to test differences in diversification dynamics between páramo and non-páramo lineages. The BiSSE method simultaneously estimates speciation and extinction rates associated with each habitat. A full BiSSE model estimates six parameters: speciation and extinction rates for páramo and non-páramo respectively, and rates of anagenetic character change from non-páramo to páramo and from páramo to non-páramo. We predict that speciation rates should be higher for páramo lineages since this represents a geologically recent habitat. Similarly, we predict that if niche space is limiting in older non-páramo habitats but not in younger páramo habitats, transition rates towards páramo from non-páramo are likely to exceed the reverse.

Our main phylogenetic analyses include specimens from multiple localities from each described species but do not include all described species of *Jamesonia* or *Eriosorus* [26], [27]. For the BiSSE analyses we sampled 1000 phylogenies randomly from the posterior distribution. For each phylogeny in turn we randomly sampled one exemplar specimen for each described species. This left 1000 phylogenies with one representative of each species in our data set and allows us to account for phylogenetic uncertainty, particularly in species delimitation. BiSSE requires that all known species are accounted for, either in the phylogeny directly or entered into the analyses as a sampling fraction. We assigned all described species that were absent from our phylogenies to either páramo (22 sampled species from 43 in total) or non-páramo (4 sampled species from 12 in total) habitat based on fieldwork observations, herbarium specimens (University Herbarium at the University of Berkeley, California) and monographs [26], [27].

We ran four BiSSE models on each phylogeny. (1) A fully parameterised state dependent model, estimating all six speciation, extinction and character state transition parameters (λ_ 0_, λ_1_, μ_0_, μ_1_, q_01_, q_10_); (2) a four parameter model with state independent speciation and extinction but state dependent character transition rates (λ, μ, q_01_, q_10_); (3) a four parameter model with state independent extinction and character transition rates but state dependent speciation (λ_ 0_, λ_1_, μ, q); and (4) a three parameter state independent model (λ, μ, q). We used maximum likelihood to fit the models. We compared the models using AICc and Akaike weights and averaged these across >1000 tree and data set combinations. We report the distribution of parameter estimates for the best-fit model across the tree sample.

### State dependent rates of morphological evolution

To test for differences in the mean and rate of evolution of pinnae length and number of leaves per frond between páramo and non-páramo lineages we used a state dependent trait evolution model [46], [47]. Specifically, the model requires that each branch in the tree is assigned to a discrete character state and then estimates the mean and rate of evolution of a continuous variable for each discrete state. The rate-heterogeneous model is then compared to a single rate model using likelihood ratio tests or AIC. To assign a character state to each branch we extracted marginal ancestral state estimates of habitat type from the BiSSE models described above. Goldberg and Igic [48] demonstrate that the BiSSE model frequently performs better than alternative maximum likelihood models (e.g. Mk2; [49], [50]), particularly where character transitions are irreversible or extremely rare. This may be the case for *Jamesonia* and *Eriosorus* where we predict transitions to páramo habitats are likely to exceed the reverse. We used the R package diversitree [45] for BiSSE analyses. The ancestral state estimation returns probabilities for each character state at each node. To each node, and to its parent branch, we assign the character state with the highest probability (pendant edges take the character state of the associated species). We then applied four alternative models: (1) a constant rate model with a mean trait value common to the two habitat types (páramo and montane), (2) a two rate model with a common overall mean, (3) a constant rate model with different means for the two habitat types and, (4) a two rate model with different means for the two habitat types. Rates of evolution can be upwardly biased if there is error or noise in the data. To account for this we simultaneously fitted Pagel's λ [42], [43] with the rates models. We compared model fit using AICc. Each model was applied to (1) pinnae length and (2) leaves per frond. We repeated all analyses, including ancestral state reconstruction, over the same 1000 sampled phylogenies used in the BiSSE analyses. Analyses of rates of morphological evolution were conducted using the R package motmot [51].

### Data availability

All data associated with this paper are available to download from FigShare http://dx.doi.org/10.6084/m9.figshare.1114946. The uploaded data includes aligned sequences, BEAST input files in xml format, a sample of 1000 trees from the posterior distribution, a Maximum Clade Credibility tree including clade posterior probabilities, and a csv file containing all trait (morphological and ecological) data.

## Results

### Phylogeny

**Figure 2:**
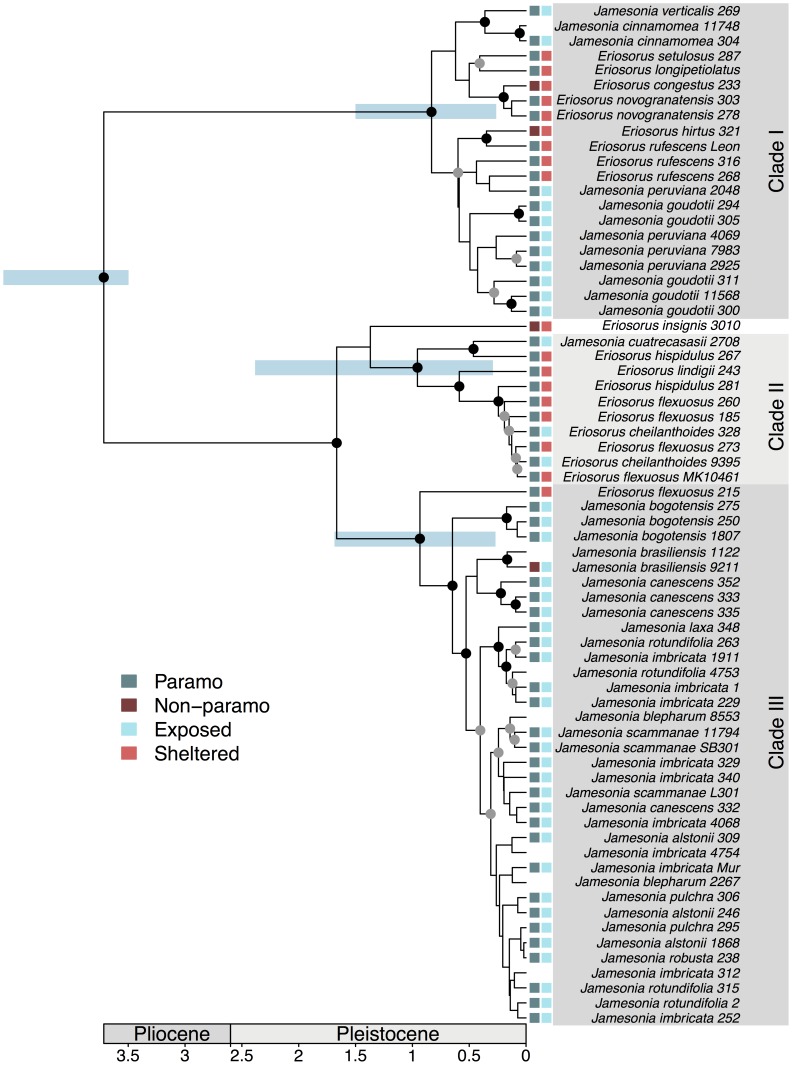
Time-calibrated phylogeny of *Jamesonia* – *Eriosorus.* Black circles indicate nodes with posterior probabilities>0.95; grey circles indicate posterior probabilities between 0.5 and 0.95. Blue bars show the 95% highest Bayesian probability densities (HPDs) for the age of select well-supported nodes. Coloured squares show presence/absence in páramo (column 1) and microhabitat type (column 2) for samples where morphological measurements were taken.

The phylogeny (Fig. 2) reveals a combination of well-supported clades deep within the tree combined with poor resolution towards the tips. We find very strong support for three previously reported [24] monophyletic groups (clades I, II, III in Fig. 2). However, whilst our analyses included multiple populations of many species, we failed to recover species monophyly in most cases (Fig. 2). Well-supported exceptions include monophyly of *J. bogotensis, J. cinnamomea*, and *J. brasiliensis*, with posterior probabilities of 1, 0.996 and 0.962. The timings of divergence are tentative because they are based on a single fossil spore and are well within the prior distribution, but are consistent with a Pleistocene diversification at a time when páramo ecosystems would have been emerging and expanding across vast areas in the Andes [3]–[5].

### Predictors of morphological evolution

**Figure 3:**
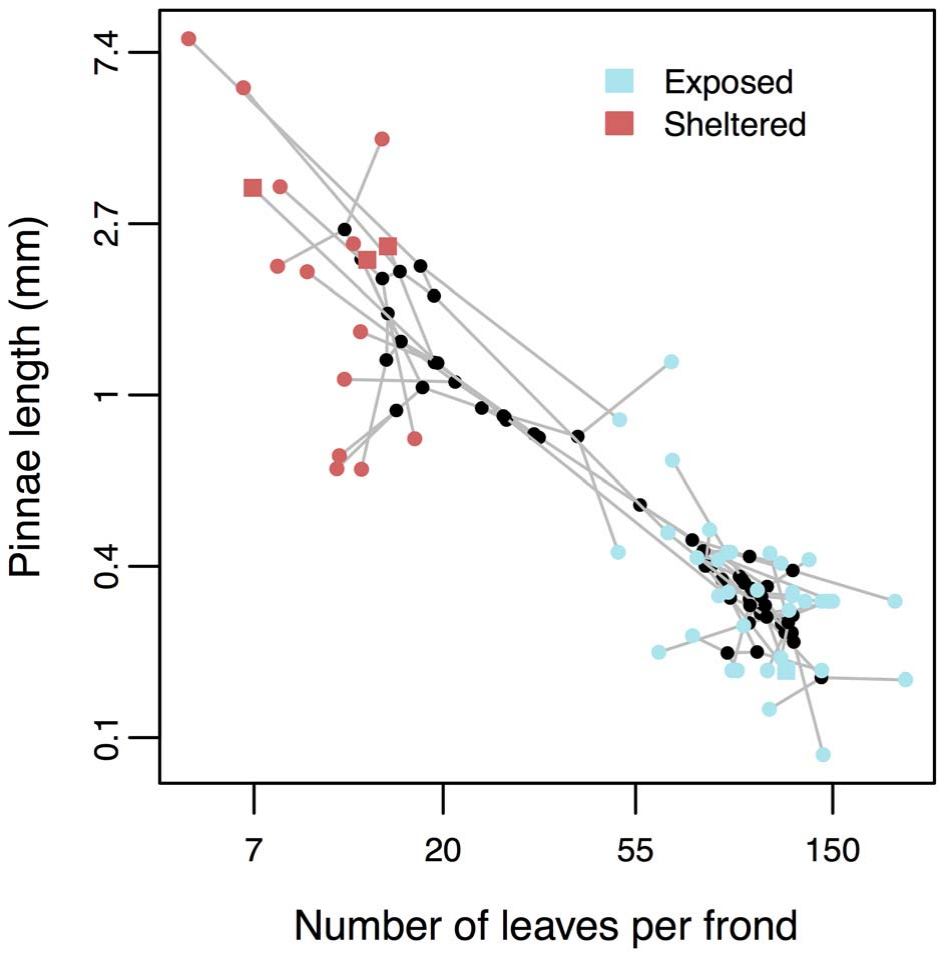
Phylomorphospace plot of log pinnae length and log number of leaves per frond. Black circles are internal nodes and are plotted as estimated ancestral states under a Brownian motion model. Grey lines are branches in the phylogeny.

We found that the two axes of leaf morphology (pinnae length and leaves per frond) form two distinct morphological clusters (Fig. 3). The two clusters correspond to habitat: taxa with long pinnae and few leaves (the *Eriosorus* morphotype) are associated with cloud forest and sheltered microhabitats within the páramo whereas taxa with short pinnae and many leaves (the *Jamesonia* morphotype) are associated with exposed (predominantly páramo) habitats.

**Figure 4:**
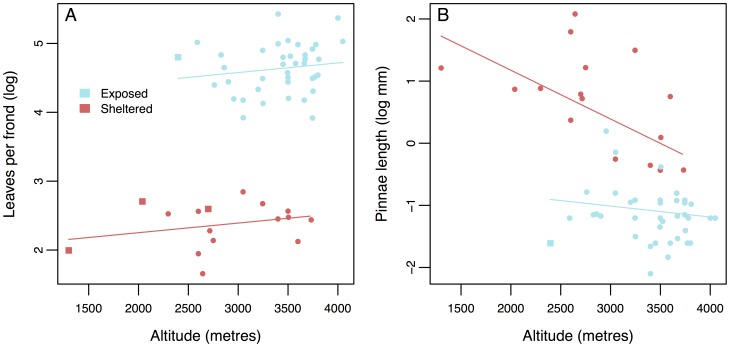
The relationship between morphology and altitude by habitat for (a) leaves per frond and (b) pinnae length. Colour of points indicates observed microhabitats. Samples from species not found in the páramo are shown as squares.

Models testing the relationship between altitude and morphology confirm these habitat dependent relationships. Two similar models are essentially indistinguishable in explaining variation in leaves per frond. One model includes both habitat and altitude but not their interaction and a simpler model includes only habitat (Table 1). In the more complex model the number of leaves per frond increases with altitude (Fig. 4a). In both models the number of leaves per frond is significantly higher among páramo species. The more complex model explains ∼90% of the variation in leaves per frond. In contrast, we find evidence of a significant interaction between habitat and altitude as predictors of pinnae length (Fig. 4b). Pinnae length declines at higher altitudes but the slope of this relationship is steeper among montane species. The shallower slope might be expected if pinnae length is approaching a lower bound in páramo species.

**Table 1:** Models of leaf morphology as a function of habitat and altitude.

(a)	Leave per frond		Pinnae length	
	ΔAICc	Akaike wts	ΔAICc	Akaike wts
Altitude	7.924	<0.001	6.073	<0.001
Habitat	0	**0.463**	2.772	0.030
Alt + Hab	0.254	0.407	0.542	0.427
Alt * Hab	2.538	0.130	**0**	**0.544**

(b)	Coefficient (est ± SE)	95^th^ percentiles
Leaves per frond		
Exposed int.	4.157 (0.344)	NA
Sheltered int.	1.972 (0.116)	NA
Altitude	1.4e-4 (10e-5)	NA
Pinnae length		
Exposed int.	−0.480 (0.823)	−0.386 – −0.683
Sheltered int.	3.225 (0.954)	2.980 – 3.575
Altitude	−1.77e-4 (2.02e-4)	−1.33e-4 – −2.16e-4
Alt:Hab	−6.08e-4 (3.02e-4)	−4.41e-4 – −7.17e-4

Note that all models fitted with interaction terms always include the same variables as main effects. (a) The fits of alternative models are compared by ΔAICc and Akaike weights averaged across 1000 trees. (b) Parameter estimates are the median estimates from pgls models fitted to 1000 trees. *Leaves per frond*: two models including habitat but no interaction term are essentially indistinguishable based on AICc, we present the results from the more complex model (Habitat + Altitude). The median maximum likelihood estimate of Pagel's lambda across trees for this model is 0 and is only non-zero in analyses with 18 from 1000 trees. Consequently, the model is equivalent to one in which phylogeny is not included, and hence there is no variation in parameter estimates attributable to variation in phylogeny. The adjusted R^2^ for the model is 0.901. *Pinnae length*: the best-fitting model including an altitude x habitat interaction term explains 56.8% of the among-species variation in pinnae length (95% quantiles for adjusted R^2^ = 0.513–0.735). The median estimate of Pagel's lambda for the interaction model across 1000 trees is 0.779 (95% quantiles = 0.000–0.957) and greater than 0.5 in analyses on 872 trees.

### BiSSE: state dependent diversification

**Figure 5:**
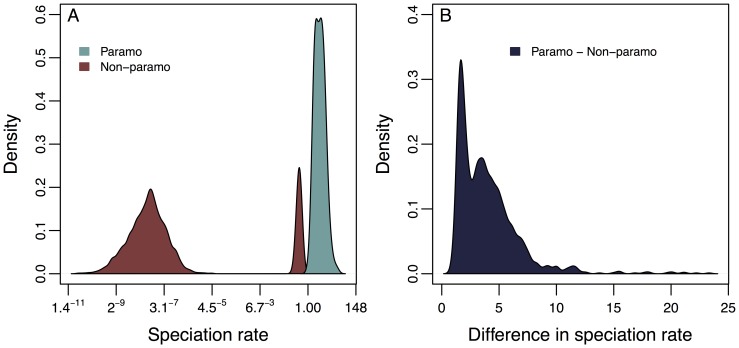
Probability densities of speciation rate estimates for the best fitting BiSSE model. Probability densities are based on the maximum likelihood parameter estimates on each of 1000 alternative phylogenies. Panel (a) shows estimates of speciation rate for páramo and non-páramo species. Extinction and transition rates do not differ in the best-fit model. Panel (b) shows the difference in speciation rates between páramo and non-páramo species plotted as páramo speciation rate – non-páramo speciation rate.

The top-ranked diversification model is one with state dependent speciation rates but state independent extinction and character transitions rates (Table 2). That is, we found no difference in transitions into and out of the páramo. Speciation rates were consistently higher for páramo species than for non-páramo species (Fig. 5a). There is a bimodal distribution of speciation rates for non-páramo species across trees. In a small subset of trees, speciation rates for non- páramo are elevated but remain lower than those for páramo species (Fig. 5b).

**Table 2:** BiSSE model comparison.

Model	ΔAICc	Akaike wts
λ_ 0_, λ_1_, μ_0_, μ_1_, q_01_, q_10_	5.910	0.036
λ, μ, q_01_, q_10_	3.359	0.196
λ_ 0_, λ_1_, μ_,_ q	0	0.616
λ, μ, q	2.878	0.152

The fits of alternative models are compared by ΔAICc and Akaike weights averaged across 1000 trees.

### State dependent rates of morphological evolution

The pattern of evolutionary convergence within habitat types was confirmed by evolutionary rate analyses. The phylogenetically corrected mean of the number of leaves per frond varies markedly with microhabitat (Table 3). However, after accounting for the possible effects of noise in the data, we found no evidence for state dependent variations in rates of evolution of leaves per frond. As with leaves per frond, pinnae length also varies with microhabitat. We also found equivocal evidence for elevated rates of evolution of pinnae length associated with exposed habitats. The Akaike weights marginally favour a state independent over a state dependent model (Table 3) but parameter estimates consistently show that rates of evolution are relatively higher among species occupying exposed habitats. Because the uncertainty in these parameter estimates is very large we suggest that these higher rates should be interpreted with caution.

**Table 3:** State dependent rates of morphological evolution.

Model	ΔAICc	Akaike wts	Mean (sheltered)	Mean (exposed)	Rate (sheltered)	Rate (exposed)
Leaves per frond						
1	33.064	<0.001	3.266		1	-
			(3.102, 3.437)			
2	29.919	<0.001	2.596		1	0.083
			(2.354, 4.389)			(0.012, 3028.817)
3	0	**0.753**	**4.254**	**2.506**	**1**	**-**
			**(4.028, 4.454)**	**(2.365–2.731)**		
4	2.344	0.247	4.204	2.431	1	0.326
			(3.888, 4.430)	(2.252, 2.678)		(<0.001, 1.591)
**Pinnae length**						
1	14.501	0.005	−0.148	-	1	-
			(−0.315, 0.116)			
2	13.634	0.004	−0.809	-	1	73
			(−1.160, 0.057)			(0.441, 4.262^4^)
3	0	**0.553**	**−0.963**	**0.501**	**1**	**-**
			**(−1.348, −0.638)**	**(0.142, 0.795)**		
4	0.409	0.438	−1.014	0.448	1	>1^5^
			(−1.323, −0.788)	(−0.125, 0.955)		(1.141,>1^5^)

The fits of alternative models for both leaves per frond and pinnae length are compared by ΔAICc and Akaike weights averaged across 1000 trees. Parameter estimates are reported as the median estimate across the tree distribution with 5^th^ and 95^th^ percentiles. Evolutionary rates are reported as relative rates.

## Discussion

While fern groups are very abundant in cloud forest, there are overall far fewer species in páramos [7], [21]. Given the proliferation of other angiosperm clades in the páramo plant hotspot [9], the lack of divergence of ferns is perhaps surprising. The *Jamesonia-Eriosorus* complex provides one counter example, where morphological adaptation to cool and exposed environments has facilitated the transition from sheltered montane habitats. Our analyses suggest that the successful colonization of páramos by the *Jamesonia-Eriosorus* complex is associated with elevated rates of speciation and morphological adaptation.

### Morphological evolution and the transition to Páramo

Morphological adaptations among *Jamesonia* and *Eriosorus* colonising the páramo are consistent with evolution as a response to a highly exposed environment. Although we find evidence that the number of leaves per frond increases and pinnae length decreases with altitude, habitat type is the main factor contributing to variation in leaf morphology (Table 1). Among lineages there are two distinct morphological clusters that correspond closely to habitat. Many leaves per frond and short pinnae characterize lineages associated with páramo. The majority of these lineages belong to the genus *Jamesonia* (with the exception of *Eriosorus cheilanthoides*). In contrast, few leaves per frond and long pinnae characterize many species of *Eriosorus* and are associated with montane or sheltered páramo habitats. The evolution of increased numbers of leaves per frond and short pinnae appears to be an example of convergent evolution in which mutliple lineages responsed to the extreme enviromental conditions in similar ways. This renders the *Jamesonia* (exposed) morphotype polyphyletic. We suggest that the well-defined exposed páramo morphology is evidence for the adaptive advantage of increased numbers of leaves per frond and short pinnae provide in extreme environmental conditions characteristic of páramo.

The effect of freezing temperatures seems to be a major factor limiting species distributions in tropical and subtropical high mountain ecosystems [52]. Plants that grow close to the ground are exposed to more drastic daily leaf temperature changes. There is a temperature gradient from the soil upwards as a result of radiant heating with significantly higher daytime temperatures but notcural freezing closer to the ground [53]. All *Jamesonia* species and *Eriosorus* species associated with exposed microhabitats have evolved erect and taller fronds (Fig. 4) with an apical frond meristem that is therefore further away from the soil compared to *Eriosorus* species in sheltered microhabitats. Under these conditions, a taller frond apical meristem likely helps to protect meristematic tissues from freezing due to extremely low nocturnal temperatures at ground level, a trait shown to be advantageous in angiosperms exhibiting tall aerial stems [54], [55]. Moreover as a consequence of increased numbers of pinnae per frond, species in microhabitats bear more sporangia, possibly allowing more spores to be dispersed.

The evolution of the exposed microhabitat morphology may also have been driven by selection for increased photosynthetic capabilities along altitudinal gradients. Previous physiological studies along an altitudinal gradient in the Andes have shown how differences in leaf temperature, influenced both by plant form and microenvironmental conditions, affect photosynthetic capacity [56]. Low temperature is a major abiotic factor limiting photosynthetic carbon acquisition ([52], [57]; but see [58] for counter examples), as well as controlling plant growth and survival in high mountain ecosystems [59]. Plants with a herbaceous habit such as *Senecio* exhibited a decrease in optimal leaf temperature for photosynthesis coupled to decreasing air temperature with increasing elevation [56]. Similar to *Senecio, Jamesonia* exhibit an herbaceous habit. A higher number of pinnae per frond could increase photosynthetic capacity, thus counteracting decreased photosynthetic rates resulting from low temperature [7] and reduced CO_2_ assimilation rates [60], [61].

Although the strongest predictor of leaf morphology is microhabitat, it is also evident that leaf morphology, particularly pinnae length, varies with altitude where shorter pinnae are associated with higher altitudes. Our sample includes both inter and intra specifc variation and the altitudinal correlation may be in part due to phenotypic plasticity within species. Common garden or reciprocal transplant experiments may help to tease apart the mechanisms driving morphological change. Sister species such as *E. cheilanthoides* and *E. flexuosus* with contrasting morphologies and habitat prefences (páramo and sheltered lower elevation sites) would be well suited to assessing whether leaf form can be altered by environmental conditions. Taken together, habitat type seems to be the dominant factor explaining variation in leaf morphology among species while evolution along altitudinal gradients may be an important factor in explaining within species variation in leaf morphology and potentially transitions to páramo habitats. First, accessions of the same species species sampled from different altitudes show a clear reduction in pinnae size with an increase in elevation in clades I and II (e.g. *E. rufescens* accessions 268 and 316, *E. novogranatensis* accessions 278 and 303, and *E. hispidulus* accessions 267 and 281; [24]). Second, high elevation montane species, such as *E. longipetiolatus* and *E. setulosus*, exhibit an intermediate morphology with highly reduced pinnae (*Jamesonia-*like pinnae). However, they have fully developed fronds rather than indeterminate growth that is typically associated with páramo species [26]. Reduction in pinnae size is associated with high elevation habitats, yet indeterminate growth is a characteristic strictly correlated with extreme and open páramo habitats. Reciprocal transplant experiments amongst habitats would help determine whether phenotypic plasticity has played a role in the evolution of leaf size. Similarly sharp ecological and morphological shifts have been reported in other neotropical plant groups such as *Huperzia* (Lycopodiaceae) where terrestrial páramo forms have evolved from montane epiphytes [23].

### Páramo radiation

Our phylogenetic analyses of the *Jamesonia-Eriosorus* clade confirm three well-supported clades inferred in previous studies [24] with multiple transitions into and out of the páramo. The *Jamesonia* ecomorph evolved independently multiple times from within different *Eriosorus* lineages (Fig. 2). The highest diversity of *Jamesonia* is found in the páramo-dominated clade III (Fig. 2), which is mostly restricted to the Northern Andes [24]. Clade I is associated with the Central Andes and contains both páramo and montane lineages whereas clade II is North Andean and dominated by montane lineages.

The páramo radiation of *Jamesonia* and *Eriosorus* shares some features with angiosperms that have radiated in páramo habitats notably including highly reduced leaves (microphylls) and pubescence. Microphyllous leaves are often xeromorphic, possibly protecting tissues from ultraviolet light and/or reducing transpiration [62], [63]. Microphyllous leaves are associated with xeric environments and seem to be present in a number of páramo taxa, particularly shrubs such as *Loricaria*, *Baccharis revoluta*, *Diplostephium revolutum*, (Asteraceae), *Aragoa cupressina* (Scrophulariaceae), and *Valeriana microphylla* (Valerianaceae; [7]). Although variable, pubescence is present in all *Jamesonia* species and is consistently present at the frond tip, presumably protecting the meristematic tissues, which could be susceptible to freezing. It has been demonstrated that dense leaf pubescence reduces transpiration and increases leaf temperature [55], [64]–[66]. Pubescence is characteristic of other genera that have presumably radiated in páramo habitats such as *Espeletia*, and it is believed to protect leaves and reduce transpiration [67]. In contrast to *Eriosorus*, all *Jamesonia* species exhibit creeping rhizomes, a feature that facilitates expansion in grasslands as shown in other páramo species such as bunchgrasses [7].

The high speciation rates in the páramo is consistent with predictions of exploitation and adatpation to new ecological niches. However, the elevated speciaton rates should be treated with caution because there are a number of caveats associated with uncertainty in the phylogeny and incomplete sampling of taxa. We found a lack of phylogenetic resolution within each of the three major clades with repeated polyphyly of traditional (i.e. taxonomic) *Jamesonia* and *Eriosorus* species. While this lack of genetic differentiation amongst the *Jamesonia-Eriosorus* complex potentially challenges the definition of traditional species as described in their monographs [24], [26], [27], it might alternatively be indicative of an ongoing radiation with continued hybridization and/or incomplete lineage sorting. Species polyphyly is particularly prevalent in clade III, which is dominated by páramo lineages. Species polyphyly could be indicative of ongoing speciation in which case we may have underestimated speciation rates. An alternative explanation for the lack of resolution may be that the molecular markers used here (*rps4* and ETS) evolve too slowly to differentiate species. However, the markers we used have previously been used for phylogenetic studies at lower taxonomic levels and *rps4* shows some of the highest substitution rates within chloroplast genes [68]. Moreover, the nuclear ETS gene has been shown to have higher substitution rates than the more commonly used ITS gene. ETS is therefore expected to be more informative than ITS in resolving phylogenetic relationships at low taxonomic levels in angiosperms [69], [70].

In this study we focused on how transitions in ecology influence the evolution of leaf morphology and patterns of diversification. We find evidence of distinct morphologies that are closely associated with transitions to exposed páramo and páramo-like habitats. However, adaptation to these exposed habitats likely involves convergent evolution of a more complex suite of traits. In exposed habitats, an increase in the number of leaves per frond likely arose as a result of retaining the apical frond meristem coupled with the retention of a ‘juvenile’ morphology (fiddlehead: furled fronds of a young fern) yet bearing sporangia. The repeated evolution of this complex of traits may have been the result of a slowdown in developmental stage at low temperatures. Comparative developmental and physiological studies are needed to better understand how the morphological adaptations associated with the páramo arose and affect survival and physiological performance in extreme and exposed habitats. Explicit tests of limits to clade diversity will require further sampling and resolution of species limits both in páramo and montane regions. Nonetheless, we find support for rapid speciation in the páramo that is consistent with diversification in a novel and often island-like habitat landscape.

## Supporting Information

Table S1 **Habitats of** ***Jamesonia*** **and** ***Eriosorus*** **species.** Habitats include: 1) super-páramo (4000–5000 m); 2) grass páramo (3500–4100 m); 3) sub-páramo (2800/3000–3500 m), and 4) montane forest (1150–2800/3000 m). Abbreviations of geographical distribution are: BO, Bolivia; BR, Brazil; CO, Colombia; CR, Costa Rica; EC, Ecuador; ME, Mexico; PA, Panama; PE, Peru; UR, Uruguay; and VE, Venezuela.
(DOC)
