# Supplementary material for: Adaptation and Convergent Evolution within the Jamesonia-Eriosorus Complex in High-Elevation Biodiverse Andean Hotspots
Source: PLoS One. 2014 Oct 23;9(10):e110618. doi: 10.1371/journal.pone.0110618 (PMC4207698; doi:10.1371/journal.pone.0110618)
Supplement: Table S1 — Habitats of Jamesonia and Eriosorus species. Habitats include: 1) super-páramo (4000–5000 m); 2) grass páramo (3500–4100 m); 3) sub-páramo (2800/3000–3500 m), and 4) montane forest (1150–2800/3000 m). Abbreviations of geographical distribution are: BO, Bolivia; BR, Brazil; CO, Colombia; CR, Costa Rica; EC, Ecuador; ME, Mexico; PA, Panama; PE, Peru; UR, Uruguay; and VE, Venezuela. (DOC) [file pone.0110618.s001.doc]

Table S1. Habitats of *Jamesonia* and *Eriosorus* species. Habitats include: 1) super-páramo (4000–5000 m); 2) grass páramo (3500–4100 m); 3) sub-páramo (2800/3000–3500 m), and 4) montane forest (1150–2800/3000 m). Abbreviations of geographical distribution are: BO, Bolivia; BR, Brazil; CO, Colombia; CR, Costa Rica; EC, Ecuador; ME, Mexico; PA, Panama; PE, Peru; UR, Uruguay; and VE, Venezuela.

| **Species** | **Habitat** | **Geographical distribution** |
| --- | --- | --- |
| **Eriosorus** |  |  |
| *E. cheilanthoides* | Sub-páramo to páramo; among rocks, at edge of boulders and open hillsides | EC, PE, BO, BR |
| *E. congestus* | Montane forest; understory, road banks, on mossy turf, shaded sites | CR |
| *E. flexuosus* | Montane forest to páramo; scrambling or climbing on shrubby growth in cloud forest and in low bordering páramos | ME, CR, PA, VE, CO, EC, PE, BOL, BR |
| *E. insignis* | Montane forest; in moist, shaded places at edge of boulders or in caves | BR |
| *E. hirsutulus* | Montane forest to sub-páramo; among rocks on peaty rock ledges and earth banks | CO |
| *E. hirtus* | Montane forest; shaded forest borders and wet places | CO, EC, BO |
| *E. hispidulus* | Montane forest; understory, road banks, bare soil, and shaded sites | CO, VE |
| *E. lindigii* | Montane forest to sub-páramo; on sandstone ledges or in shade at base of boulders | CO |
| *E.* *longipetiolatus* | Montane forest to sub-páramo; on rocks and wet places at forest borders | CO |
| *E. novogranatensis* | Montane forest to sub-páramo; climbing on steep mossy banks or forest borders | CO, EC |
| *E. rufescens* | Montane forest to páramo; on shaded crevices of bluffs, and earth banks, or in caves | VE, CO, EC, PE, BO |
| *E. setulosus* | Montane forest to sub-páramo; on mossy banks at forest borders | CO, EC |
| **Species** | **Habitat** | **Geographical distribution** |
| **Jamesonia** |  |  |
| *J. alstonii* | Sub-páramo to páramo; on rocks among mossy turf and lichens | ME, CR, CO, EC, PE, BO |
| *J. brasiliensis* | Páramo; among grasses or associated with vegetation | BO, BR |
| *J. bogotensis* | Sub-páramo to super-páramo; among rocks in very exposed areas such as cliffs and edge of boulders with superficial rhizomes | CO |
| *J. canescens* | Páramo to super-páramo; among grasses on dry slopes | CO, VE |
| *J. cinnamomea* | Páramo to super-páramo; among rocks and/or bare soil, tolerates ice | CO, EC, PE |
| *J. cuatrecasasii* | Páramo to super-páramo; among grasses on very exposed slopes and cliffs | CO, VE |
| *J. goudotii* | Páramo to super-páramo; on bare soil or among dense vegetation at edge of forest | CO, EC, PE |
| *J.* *imbricata* | Páramo to super-páramo; among dense grasses, often with deeply embedded rhizomes | CR, CO, VE, EC, PE |
| *J. laxa* | Páramo; among dense vegetation at edge of forest | VE |
| *J.* *peruviana* | Páramo; among grasses in boggy places | PE, BO |
| *J.* *pulchra* | Páramo; in open fields and grass meadows | CO, EC |
| *J.* *rotundifolia* | Sub-páramo to páramo; among dwarf shrubs and other vegetation, edge of forest | CR, CO, EC, PE |
| *J.* *scammanae* | Sub-páramo to páramo; in open fields among grasses | CR, CO |
| *J.* *verticalis* | Sub-páramo; among small bushes and other vegetation, forest edges | CO, EC |
